# Cinnamaldehyde attenuates streptozocin-induced diabetic osteoporosis in a rat model by modulating netrin-1/DCC-UNC5B signal transduction

**DOI:** 10.3389/fphar.2024.1367806

**Published:** 2024-04-02

**Authors:** Songjie Ji, Bingjia Zhao, Yuan Gao, Jun Xie, Huijun Han, Qunli Wu, Dan Yang

**Affiliations:** ^1^ Department of Orthopaedic Surgery, Beijing Jishuitan Hospital, Capital Medical University, Beijing, China; ^2^ Department of Joint Surgery, Beijing Jishuitan Guizhou Hospital, Guiyang, China; ^3^ Department of Traditional Chinese Medicine, Peking Union Medical College Hospital, Peking Union Medical College, Translational Medicine Center, Chinese Academy of Medical Sciences, Beijing, China; ^4^ Department of Epidemiology and Biostatistics, Institute of Basic Medical Sciences, Chinese Academy of Medical Sciences and School of Basic Medicine, Peking Union Medical College, Beijing, China

**Keywords:** cinnamaldehyde, diabetic osteoporosis, netrin-1, streptozocin, TRAP

## Abstract

**Background:** Cinnamaldehyde (CMD) is a major functional component of *Cinnamomum verum* and has shown treatment effects against diverse bone diseases. This study aimed to assess the anti-diabetic osteoporosis (DOP) potential of diabetes mellitus (DM) and to explore the underlying mechanism driving the activity of CMD.

**Methods:** A DOP model was induced via an intraperitoneal injection of streptozocin (STZ) into Sprague–Dawley rats, and then two different doses of CMD were administered to the rats. The effects of CMD on the strength, remodeling activity, and histological structure of the bones were assessed. Changes in the netrin-1 related pathways also were detected to elucidate the mechanism of the anti-DOP activity by CMD.

**Results:** CMD had no significant effect on the body weight or blood glucose level of the model rats. However, the data showed that CMD improved the bone strength and bone remodeling activity as well as attenuating the bone structure destruction in the DOP rats in a dose-dependent manner. The expression of netrin-1, DCC, UNC5B, RANKL, and OPG was suppressed, while the expression of TGF-β1, cathepsin K, TRAP, and RANK was induced by the STZ injection. CMD administration restored the expression of all of these indicators at both the mRNA and protein levels, indicating that the osteoclast activity was inhibited by CMD.

**Conclusion:** The current study demonstrated that CMD effectively attenuated bone impairments associated with DM in a STZ-induced DOP rat model, and the anti-DOP effects of CMD were associated with the modulation of netrin-1/DCC/UNC5B signal transduction.

## 1 Introduction

Osteoporosis is a chronic skeletal disorder that is characterized by a decreased bone mass and destruction of the bone tissue microarchitecture (Gortázar and Ardura, 2020; Moseley et al., 2021; He et al., 2022). Based on an investigation carried out in 2016, more than 6.6% of men and 22.1% of women older than 50 years old in the total European population suffer from impairments associated with osteoporosis. Currently, the altered molecular networks of the skeletal system associated with diabetes mellitus (DM) have been demonstrated as a major factor leading to a weakened effect on bone quality and strength (Ali et al., 2022). The incidence of diabetic osteoporosis (DOP) depends on a number of factors, such as obesity, the type diabetes, as well as the type of anti-DM therapy applied. Moreover, the incidence of DOP has kept increasing in recent years (Ali et al., 2022) and made the disease a common complication associated with DM. Therefore, it is necessary to explore novel molecular targets for developing effective therapies against DOP among patients suffering from DM.

The axon-guiding netrins are a well-characterized family of bone destruction modulators (Enoki et al., 2014; Mediero et al., 2015; Maruyama et al., 2016; Mediero et al., 2016), which are ubiquitously expressed in vertebrates and are involved in directing cell migration and adhesion (Lai Wing Sun et al., 2011). Netrin-1 underlies the biological functions of the other netrin family members and defines the functions of the netrin family (Cirulli and Yebra, 2007). The opposing functions of netrins are determined by the expression of the netrin receptors uncoordinated 5 (UNC5) and deleted in colorectal cancer (DCC): the former factor mediates the repulsion of neural axons, whereas the expression of the latter factor results in neuronal attraction (Cirulli and Yebra, 2007). Regarding the interaction between netrin-1 and DCC or UNC5 in bone disorders, netrin-1 is highly expressed in the synovial fluid of rheumatoid arthritis patients, and activation of the netrin-1/UNC5B axis has been shown to prevent bone destruction (Maruyama et al., 2016). Furthermore, DCC has been demonstrated to be expressed in a CD166-positive subpopulation of chondrocytes in human osteoarthritic cartilage (Bosserhoff et al., 2014). Additionally, the activation of netrin-1 also contributed to the upregulation of OPG and TGF-β1 as well as the downregulation of TRAP, RANKL, and cathepsin K. Generally, the upregulation of TRAP activity is a well-characterized phenotypic marker for osteoclasts (Tanaka et al., 2005). Regarding the OPG/RANKL/RANK pathway, RANKL is a transmembrane protein ubiquitously expressed in the cell membrane of osteoblasts and activates the RANK receptor to form new bone (Rodan and Martin, 1981; Boyce and Xing, 2008). The interaction between RANKL and RANK can be blocked by OPG, which contributes to the inhibited generation of osteoclasts (Nagy and Penninger, 2015). Moreover, TGFβ1 can inhibit human RANKL-induced osteoclastogenesis via suppressing the expression of nuclear factor of activated T cells 1 (Tokunaga et al., 2020), while cathepsin K is a lysosomal cysteine protease with a high expression in osteoclasts (Janiszewski et al., 2023). Even though netrin-1 along with its downstream effectors is well known to be involved in the modulation of bone metabolism and contributes to the progression of various bone disorders, their roles in the development of DOP are not well understood. Therefore, it is reasonable to assess whether the specific modulation of netrin-1 and its downstream effectors can serve as a promising strategy for treating DOP and other bone disorders.

**FIGURE 1:**
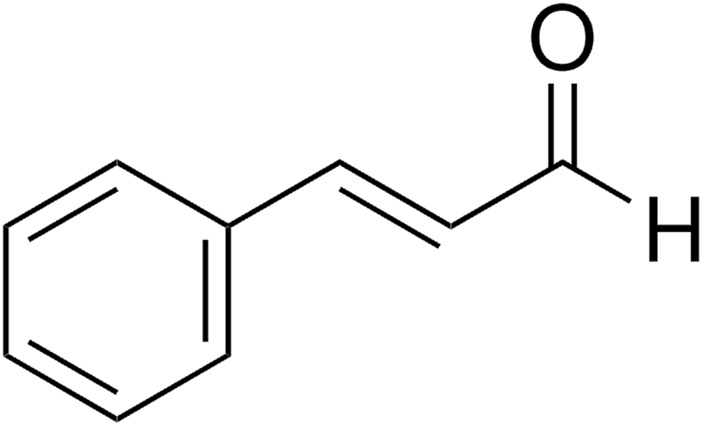
Structure of cinnamaldehyde.

There has been emerging interest in the potential of traditional Chinese medicine (TCM) for the treatment to manage various types of bone disorders (Zhang et al., 2016). Cinnamaldehyde (CMD) (C_6_H_5_CH = CHCHO) (Figure 1) is an aldehydic component extracted from the bark of *Cinnamomum verum* J. Presl. *C. cortex* is a well-known Chinese herb that is reported to have immunomodulatory, antioxidant, and neurotrophic activities (Liu et al., 2021). Moreover, this herb has been employed to treat osteoporosis in people in China for years (Gao et al., 2013). In ovariectomized rats, the administration of CMD has been shown to promote osteogenesis (Tsuji-Naito, 2008), and the combined treatment with both CMD and parathyroid hormone (PTH) also has been demonstrated to enhance the therapeutic effect of PTH on glucocorticoid-induced osteoporosis in rats (Wu et al., 2018a). Based on the study by Ji et al., CMD also contributes to nerve regeneration by modulating the netrin-1/DCC axis, which indicates that this compound has a potential regulatory effect on netrin-1 (Ji et al., 2016). Collectively, the relationships between DOP, CMD, and netrin-1/DCC/UNC5B pathways are worthy of exploration, which will provide valuable information for explaining the mechanism underlying the potential treating effects of CMD against DOP.

Therefore, in the current study, CMD was employed to manage streptozocin (STZ)-induced DOP symptoms in rats. The mechanism underlying the anti-DOP activity of CMD was explored by focusing on its effects on the activity of netrin-1 and its downstream effectors.

## 2 Materials and methods

### 2.1 Chemical and antibody information

CMD (purity: ≥95%) was purchased from Sigma-Aldrich (China). Antibodies against netrin-1 (ab126729), UNC5B (ab189914), and RANK (ab305233) were purchased from Abcam (China). Antibody against DCC (19,123-1-ap) was purchased from Proteintech (China). Antibodies against OPG (sc-390518), RANKL (sc-377079), TGF-β1 (sc-130348), and cathepsin K (sc-48353) were purchased from Santa Cruz Biotechnology (China). Antibody against TRAP (GTX30018) was purchased from GeneTex (China). Antibody against GAPDH was purchased from Cell Signaling Technology (China).

### 2.2 Animals and study design

Eight-week-old male Sprague–Dawley (SD) rats (Certificate Number: SCXK 2013-0034) were provided by Beijing Vital River Laboratory Animal Technology Co., Ltd. (Beijing, China) and were housed under standard conditions. All animal experiments were performed under the approval by the Committee on Animal Care and Use of Peking Union Medical College Hospital, Chinese Academy of Medical Sciences and Peking Union Medical College.

Thirty-two SD rats were randomly divided into the following four groups (eight per group) to assess the effect of CMD on DOP symptoms: Control group, healthy SD rats received a suspension of 0.5% carboxymethylcellulose (10 mL/kg/day, by oral gavage) for 12 weeks; DOP group, rats were injected with STZ intraperitoneally (60 mg/kg body weight) and received a suspension of 0.5% carboxymethylcellulose (10 mL/kg/day, by oral gavage) for 12 weeks after the STZ injection [the hyperglycemic condition in the DOP group was determined by measuring the blood glucose level at 72 h after the STZ injection using a glucometer (a blood glucose level >16.7 mmol/L was maintained for 12 weeks to induce osteoporosis)]; DOP + CMD20 group, DOP rats were gavaged daily with 20 mg/kg/day CMD (purity≥95% dissolved in carboxymethylcellulose, Sigma-Aldrich, China) for 12 weeks based on the previous studies (Wu et al., 2018a; Mateen et al., 2019); and DOP + CMD40 group, DOP rats were gavaged daily with 40 mg/kg/day CMD for 12 weeks based on the previous studies (Wu et al., 2018a; Mateen et al., 2019). The changes in body weight and blood glucose levels were monitored in all rats every 4 weeks throughout the 12-week period.

### 2.3 Three-point bending test and micro-computed tomography (micro-CT)

The effect of CMD on the femoral strength of the DOP rats was detected with the three-point bending mechanical test using an electronic universal testing machine (AG-X plus, Shimadzu, Japan). Briefly, the rats were sacrificed and the femurs were collected. With the span of the fulcrum being adjusted to 20 mm, the femoral samples were loaded in the same position at a deformation rate of 1 mm/min until a fracture occurred. The ultimate load data were analyzed with the bundled software (TRAPEZIUMX, Shimadzu, Japan).

The microstructure of the femur was evaluated by detecting the bone surface/bone volume ratio (BS/BV), bone volume/tissue volume ratio (BV/TV), trabecular thickness (Tb. Th), trabecular number (Tb. N), and trabecular separation (Tb. Sp) using a SkyScan 1076 micro-CT scanner (viva CT40, Skyscan, Belgium).

### 2.4 Hematoxylin–eosin (H&E) staining

Histological changes in trabeculae were determined using H&E staining. Briefly, fresh bones tissues were fixed, soaked in serial ethanol solutions, dehydrated, and cross-sectioned. Then all of the 5-µm-thick sections were stained with H&E. The images were captured using a microscope at magnifications of ×100 and 400×.

### 2.5 Reverse transcription quantitative polymerase chain reaction (RT-qPCR)

Total RNA was extracted, and the concentration of RNA was measured with a spectrophotometer. Templates of cDNA were achieved using a reverse transcriptase kit (Fermentas, United States). The primer information is shown in Table 1. Amplification was performed according to standard methods using a Bio-Rad CFX Manager system, and the relative expression levels of target genes were calculated according to the 2^−(ΔΔCt)^ method.

**TABLE 1:** Primer information.

Gene	Direction (5′-3′)	Sequence
TRAP	Forward	GGC​TAC​CTA​CGC​TTT​CAC​TAT​G
	Reverse	TTT​CCA​GAG​GCT​TCC​ACA​TAC
UNC5B	Forward	CGA​CCC​TAA​AAG​CCG​CCC​C
	Reverse	GGG​ATC​TTG​TCG​GCA​GAG​TCC
DCC	Forward	ACA​TCC​GAC​GTT​CGG​CTT​T
	Reverse	TGA​TTT​TCC​CAT​TGG​CTT​CC
Netrin-1	Forward	AGA​GTT​TGT​GGA​TCC​GTT​CG
	Reverse	TTC​TTG​CAC​TTG​CCC​TTC​TT

### 2.6 Western blot assay

Total proteins were extracted using radioimmunoprecipitation assay buffer (Beyotime, China). Next, 20–30 μg of protein sample was separated by sodium dodecyl sulfate polyacrylamide gel electrophoresis, and then the proteins were transferred to a polyvinylidene difluoride membrane. Primary antibodies against netrin-1, UNC5B, DCC, TRAP, OPG, RANK, RANKL, TGF-β1, cathepsin K, and GAPDH were first incubated with the membranes and then with the secondary antibodies for 1 h at room temperature. The relative expression levels (control represented as 1) of the target proteins were calculated using Image Lab software, version 5.1 (Bio-Rad Laboratories Inc., United States).

### 2.7 Immunohistochemistry (IHC)

For IHC analysis, paraffin-embedded bone sections were hydrated, fixed, and then incubated with primary antibody against TRAP at 37°C for 30 min before incubation at 4°C overnight. Afterwards, secondary antibody (1:200) was incubated with the sections at 37°C for 30 min. After another incubation with horseradish peroxidase-labeled avidin and 3,3′-diaminobenzidine, the sections were restained using hematoxylin, dehydrated, and images were captured using an Olympus BX43 light microscope (Olympus, Japan) at magnifications of ×100 and 400×.

### 2.8 Statistical analysis

All data were expressed as the mean ± standard deviation. One-way analysis of variance with Dunnett’s *post hoc* test and the Student’s t-test or Kruskal Wallis test were conducted using GraphPad Prism software, and a two-tailed *p*-value < 0.05 indicated a significant difference.

## 3 Results

### 3.1 CMD does not significantly affect the body weight or blood glucose level of DOP rats

**FIGURE 2:**
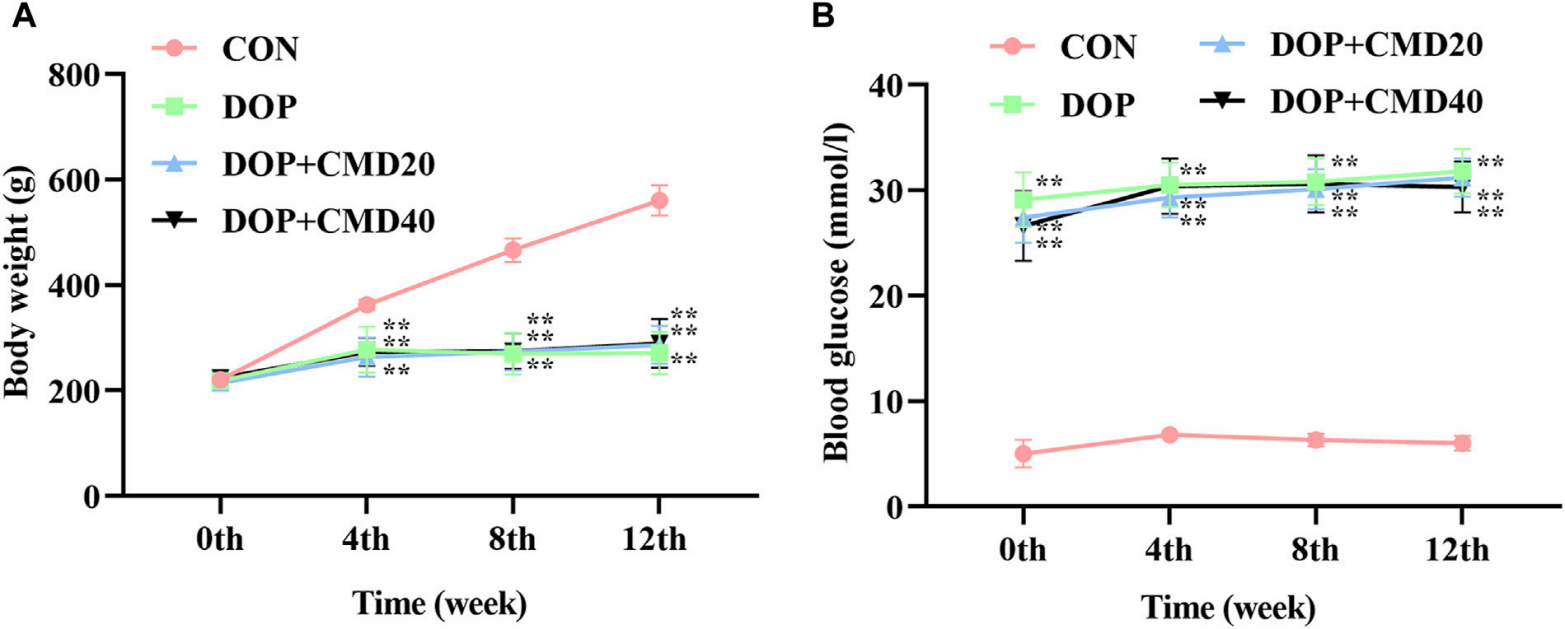
Effects of CMD on the body weight and blood glucose level of DOP rats. DOP symptoms were induced via an intraperitoneal injection of STZ (60 mg/kg body weight) and then treated with CMD (20 and 40 mg/kg body weight). **(A)** Body weight results. **(B)** Blood glucose level results. Control group, healthy rats; DOP group, rats with DOP symptoms; CMD group, DOP rats treated with CMD. “**” represents statistical significance, *p* < 0.05.

The effects of CMD on DOP rats were first assessed by measuring the changes in the body weight and blood glucose level. As shown in Figure 2A, the establishment of the DOP model significantly reduced the increase in body weight of the rats compared with that of the control group (*p* < 0.05). The administration of CMD at either dose had little effect on the loss of body weight (Figure 2A) (*p* > 0.05). Regarding the effect of CMD on the blood glucose level, a similar effect was observed (Figure 2B): the STZ treatment significantly increased the blood glucose level in the DOP rats compared with that of the control rats, and the administration of CMD at either dose did not restore the blood glucose to a relatively normal level, even after the 12-week treatment (Figure 2B). The significantly high blood glucose level confirmed the establishment of the DM model via the injection of STZ. Additionally, given that CMD had no effect on the body weight or blood glucose level in the DOP rats, any potential effects of CMD on DOP should not be attributed to the attenuation of hyperglycemia.

### 3.2 CMD improves bone strength and bone remodeling activity in DOP rats

**FIGURE 3:**
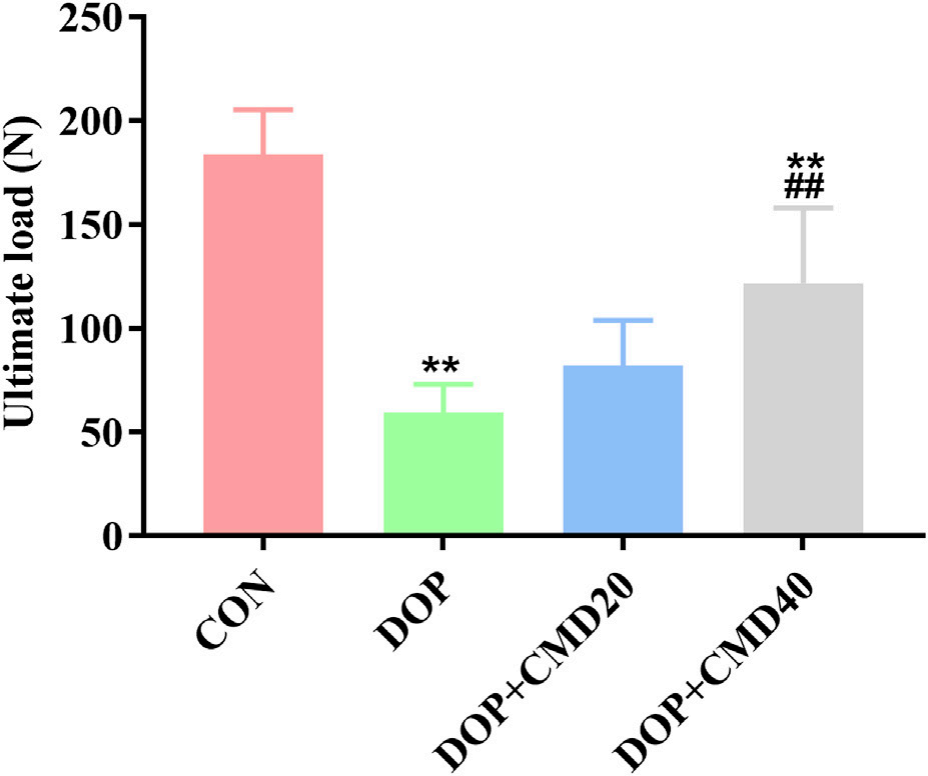
Effects of CMD on the bone strength of DOP rats. DOP symptoms were induced via an intraperitoneal injection of STZ (60 mg/kg body) and then treated with CMD (20 and 40 mg/kg body weight). The bone strength results are shown. Control group, healthy rats; DOP group, rats with DOP symptoms; CMD group, DOP rats treated with CMD. *p < 0.05 vs. CON; ^#^ *p* < 0.05 vs. DOP; ^&^ *p* < 0.05 vs. DOP + CMD20.

**FIGURE 4:**
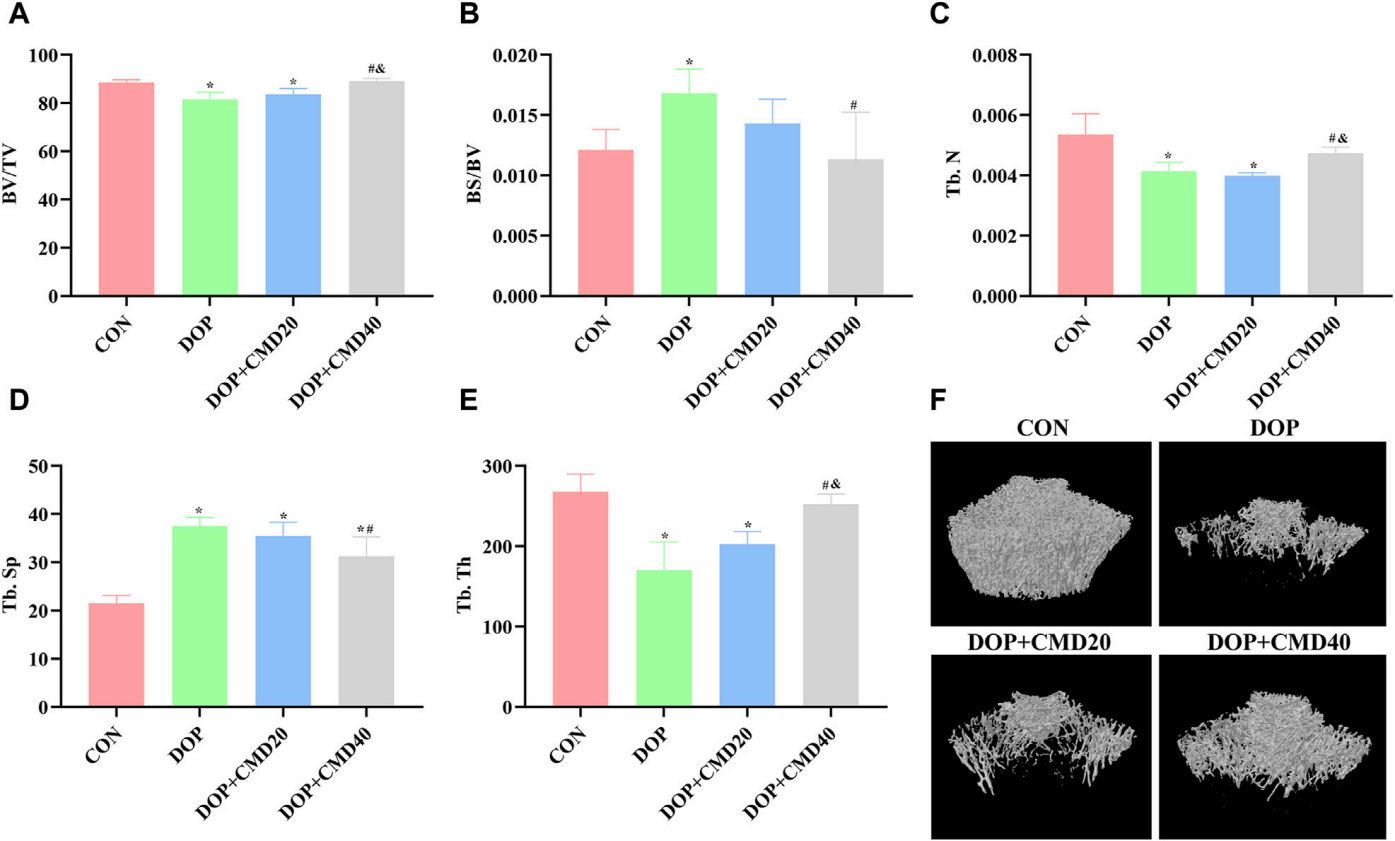
Effects of CMD on the bone remodeling activity of DOP rats. DOP symptoms were induced via an intraperitoneal injection of STZ (60 mg/kg body weight) and then treated with CMD (20 and 40 mg/kg body weight). The bone structural histomorphometric parameter results are shown. **(A)** BV/TV results. **(B)** BS/BV results. **(C)** Tb. N results. **(D)** Tb. Sp results. **(E)** Tb. Th results. **(F)** micro-CT images. Control group, healthy rats; DOP group, rats with DOP symptoms; CMD group, DOP rats treated with CMD. *p < 0.05 vs. CON; ^#^ *p* < 0.05 vs. DOP; ^&^ *p* < 0.05 vs. DOP + CMD20. BS/BV, bone surface/bone volume; BS/BV, trabecular bone surface/bone volume; BV/TV, bone volume/tissue volume; Tb. Th, trabecular thickness; Tb. N, trabecular number; Tb. Sp; trabecular separation.

To further verify the anti-DOP effects of CMD, the changes in bone strength and bone remodeling activity of DOP rats were detected by the three-point bending test and micro-CT. Based on the results of the three-point bending test, in the DOP group, the femur showed a lower ultimate load value than that of the control group, indicating a lower mechanical strength (Figure 3) (*p* < 0.05). After the CMD treatment at either dose, the mechanical strength was restored (*p* < 0.05) (Figure 3). However, compared with the DOP group, only the CMD treatment at 40 mg/kg showed a statistically significant difference in the mechanical strength (*p* < 0.05); while the CMD treatment at 20 mg/kg did not significantly improve the mechanical strength (Figure 3) (*p* > 0.05). These findings indicated a dose-dependent effect of CMD on the skeletal system. Regarding changes in the bone remodeling activity, the micro-CT results showed that CMD administration at either dose reversed the effects of STZ treatment on histomorphometric indexes by increasing the levels of BV/TV, Tb. N, and Tb. Th, while decreasing the levels Tb. Sp and BS/BV in the DOP rats (Figures 4A–E). The three-dimensional images of the coronal plane obtained by micro-CT are shown in Figure 4F, which further supported the histomorphometric data. Similar to the findings of the three-point bending tests, the effects of CMD at 40 mg/kg were still stronger than those of CMD at 20 mg/kg, indicating that the bone protective effects of CMD depended on the dose: a lower dose of CMD might have little effect against bone impairments induced by STZ or other treatments. Based on the results of the three-point bending tests and micro-CT, H&E staining, IHC, Western blot, and RT-qPCR assays were performed with the control, DOP, and DOP + CMD40 groups.

### 3.3 CMD improves the histological structure of the femur in DOP rats

**FIGURE 5:**
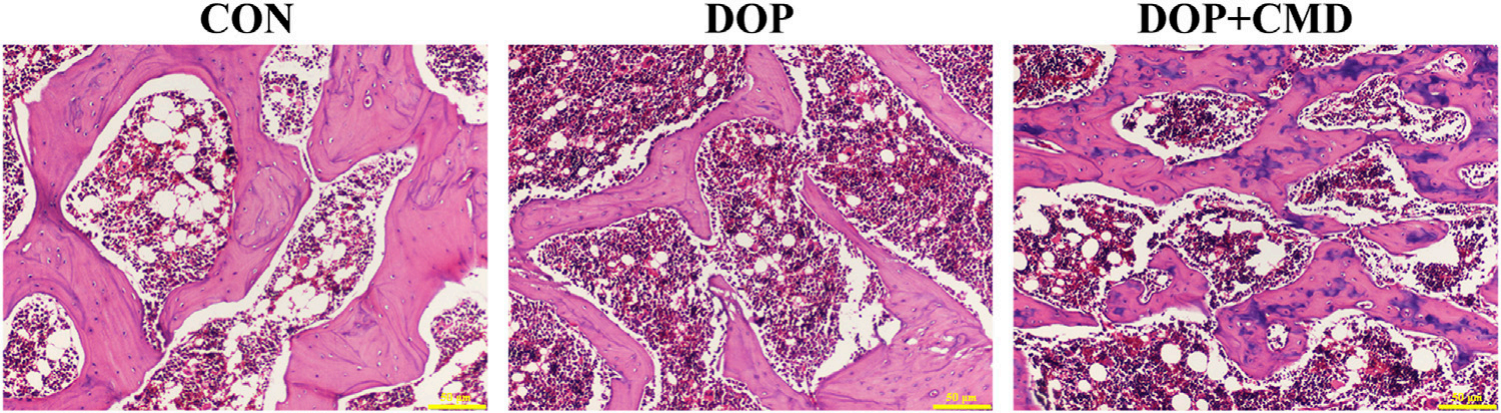
Effects of CMD on the histological structures of the femur and mandible of DOP rats. DOP symptoms were induced via an intraperitoneal injection of STZ (60 mg/kg body weight) and then treated with CMD (40 mg/kg body weight). The representative images of H&E detection. Control group, healthy rats; DOP group, rats with DOP symptoms; CMD group, DOP rats treated with CMD. Scale bar, 50 μm.

The histological changes in femur tissues of rats in the different groups were detected using H&E staining, which directly demonstrated the bone protective effects of CMD. As shown in Figure 5, thinner cortical bone tissues were observed in the femur tissues in the DOP rats compared to those of the control group. However, for the DOP rats administered with CMD at 40 mg/kg, an increased cortical bone thickness of the femur was observed (Figure 5). Moreover, the thickness was even comparable to that of the control group, further supporting the bone protective effects of CMD. Correspondingly, in the DOP group, the trabecular bone of the femur was remarkably thinned and the structure was obviously separated (Figure 5). In contrast, in the control group, the trabecular bone structure of the femur was dense and neatly arranged (Figure 5). Similar to the structural changes in the cortical bone, the impairments of the trabecular bone structure were ameliorated by the administration of CMD at 40 mg/kg; increases in Tb. N and Tb. Th were observed in the femurs of the CMD group (Figure 5). Furthermore, the Tb. Sp in the CMD groups was also much thinner than that of the DOP group (Figure 5). The morphological changes in the femurs of the DOP rats before and after CMD administration solidly supported the results of the three-point bending tests and micro-CT, further indicating that CMD treatment effectively alleviated the destruction of the skeletal system induced by STZ in the DOP rats. Therefore, there is evidence that CMD can be employed as a potential treatment strategy against DOP or other types of osteoporosis.

### 3.4 CMD activates the netrin-1/DCC/UNC5B pathway and suppresses TRAP in DOP rats

**FIGURE 6:**
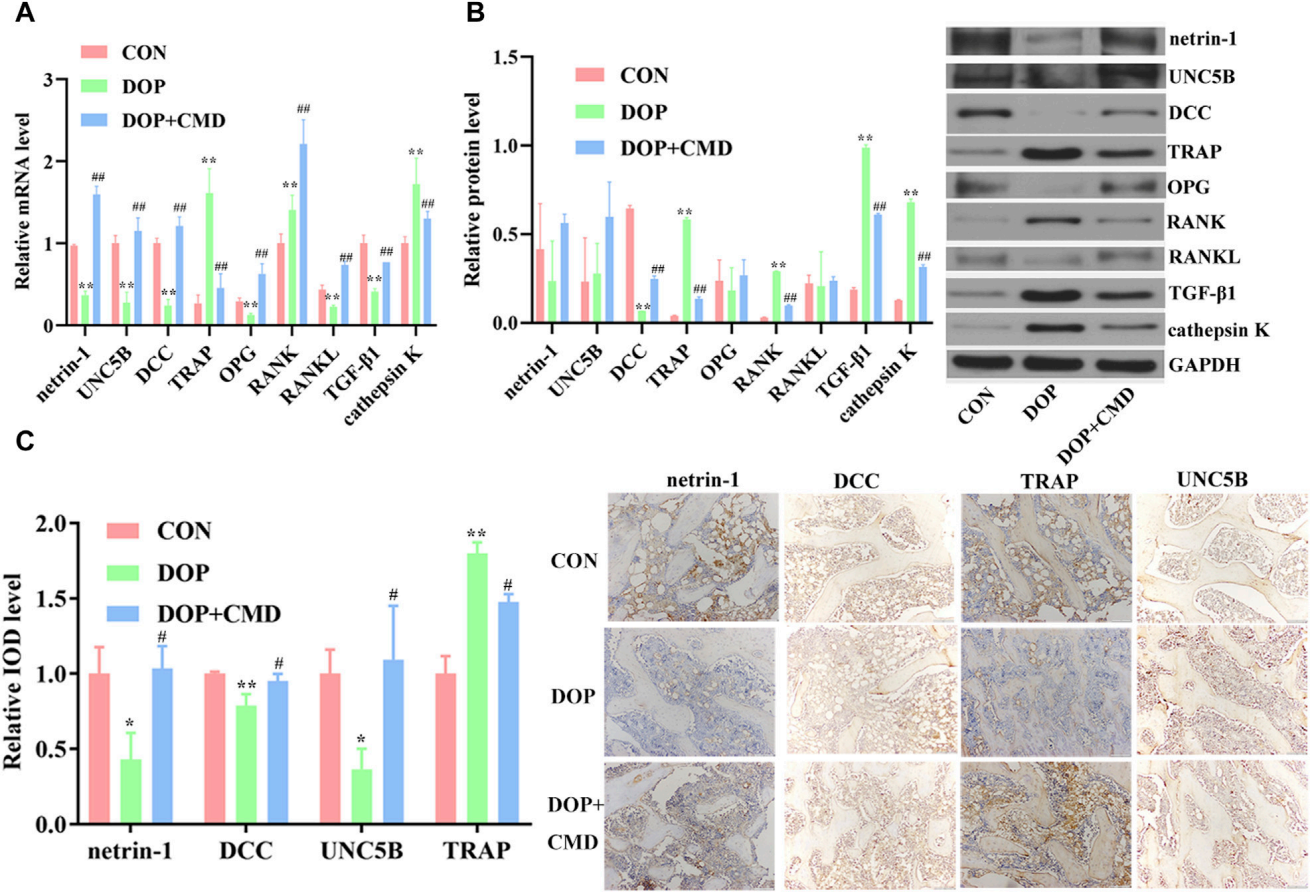
Effects of CMD on the activities of netrin-1/DCC/UNC5B, TRAP, the OPG/RANK/RANKL axis, TGF-β1, and cathepsin K in CMD-treated DOP rats. DOP symptoms were induced via an intraperitoneal injection of STZ (60 mg/kg body weight) and then treated with CMD (40 mg/kg body weight). **(A)** Representative images and results of the RT-qPCR assay. **(B)** Representative images and results of the Western blot assay. **(C)** Representative images and results of IHC detection. **p < 0.01 vs. CON group; ^##^ *p* < 0.01 vs. DOP group. Magnification, ×100.

To reveal the potential mechanism mediating the anti-DOP activity of CMD, changes in the netrin-1/DCC/UNC5B pathway were detected. Based on the RT-qPCR assay results, the mRNA expression levels of netrin-1, DCC, and UNC5B were significantly lower in the DOP group than in the control group (*p* < 0.05), which contributed to the inhibition of OPG, RANKL, and TGF-β1 (Figure 6A). On the contrary, the mRNA expression levels of TRAP, RANK, and cathepsin K were upregulated by STZ treatment (Figure 6A) (*p* < 0.05). After the administration of CMD at 40 mg/kg, the mRNA expression levels of these biomarkers were restored (Figure 6A). Similar results were achieved by Western blot assays; compared with the control rats, the establishment of the DOP model suppressed the protein expression of netrin-1, DCC, UNC5B, OPG, and RANKL in bone tissues, while the protein expression and distribution of TRAP, RANK, TGF-β1, and cathepsin K were increased (Figure 6B). The only inconsistency between the RT-qPCR and Western blot assays was the results of TGF-β1 expression, which was suppressed based on the mRNA level but was induced based on the protein level. The mechanism driving this discrepancy needs to be further explored. After the CMD treatment, the protein expression pattern in the DOP rats was reversed in an identical pattern to that of mRNA. Moreover, the current study also detected the distribution of netrin-1, DCC, TRAP, and UNC5B by IHC. The findings revealed that the STZ treatment suppressed the distribution of netrin-1, DCC, and UNC5B, while it expanded the distribution TRAP (Figure 6C), indicating that the activity of osteoclasts in the DOP group was induced after the STZ treatment. However, the expression status of the three proteins was also reversed by CMD, further confirming the protective effects of CMD against DOP.

## 4 Discussion

As a major functional component of *C. verum*, CMD has shown protective effects against various types of bone diseases in previous studies (Wu et al., 2018a; Wu et al., 2018b). In the current study, we attempted to assess the effects of CMD in the treatment of DOP, a common complication of DM. The administrating doses of CMD were 20 mg/kg/day and 40 mg/kg/day respectively, which were selected based on the previous works that CMD ranging from 20 mg/kg/day to 50 mg/kg/day had protective effects against bone disorders (Wu et al., 2018a; Mateen et al., 2019). Our data demonstrated that CMD effectively improved the strength, remodeling activity, and histological structure of bones in DOP rats; however, it did not have a significant influence on the body weight or blood glucose level. Therefore, these data solidly support our hypothesis that CMD can attenuate impairments associated with DOP. The current study also explored the potential mechanism underlying the anti-DOP effects of CMD. The administration of CMD activated the netrin-1/DCC/UNC5B and TGF-β1 pathways as well as inhibited the OPG/RANKL and cathepsin K pathways in DOP rats, providing a preliminary explanation for the activity of CMD.

Netrin-1 is a typical member of the netrin family, the expression of which prevents leukocyte arrest and tissue entry in the endothelium (van Gils et al., 2013). The protein factor may also contribute to the attenuation of inflammation-related disorders in the skeletal system. For example, Maruyama et al. have revealed that netrin-1 exerts a prophylactic function against bone degradation in a mouse model (Maruyama et al., 2016). Another study performed by Medieor et al. has demonstrated that netrin-1 and its receptor UNC5B could serve as molecular targets for the management of inflammatory arthritis and that the effects were associated with the restored level of cathepsin K (Mediero et al., 2016). UNC5 and DCC are two typical netrin receptors that are involved in mediating the attraction and repulsion of neural axons by members of the netrin family. Regarding their roles in bone disorders, UNC5B, as a downstream effector of netrin-1, has been shown to prevent bone destruction (Maruyama et al., 2016), and DCC has been reported to be expressed in a CD166-positive subpopulation of chondrocytes in human osteoarthritic cartilage (Bosserhoff et al., 2014). Thus, the specific modulation of DCC and UNC5B via netrin-1 may serve as a promising strategy to treat bone disorders. The current study detected changes in netrin-1/DCC/UNC5B pathways in CMD-treated DOP rats, and the data showed that the administration of CMD restored the levels of netrin-1, DCC, UNC5B, and OPG, while it suppressed the expression of TRAP, RANKL, and RANK, solidly indicating the inhibited osteoclast activity in DOP rats.

The modulation of netrin-1-mediatd pathways by CMD provide additional information for the mechanism underlying the anti-DOP function of CMD. In previous studies regarding the treatment effects of CMD against various bone disorders, few molecular mechanisms have been proposed. For instance, Wu et al. paid more attention to the effects of CMD on the differentiation of osteoblasts (Wu et al., 2018a; Wu et al., 2018b), without explaining the molecular pathways mediating the effects. Thus, the findings of the current study can serve as a supplement to these previous studies regarding the treatment effects of CMD against bone disorders other than DOP. In fact, the potential modulatory effects of CMD on the netrin-1/DCC/UNC5B pathway have been reported in the nervous system. For example, Ji et al. have reported that You-Gui pills, with *C. verum* as one of the major ingredients, promoted nerve regeneration by regulating the netrin-1/DCC pathway (Ji et al., 2016). This effect was further supported by our data in the DOP model, indicating that modulation of the netrin-1/DCC/UNC5B pathway by CMD may universally exist in different systems. Together with the modulation of TRAP and the OPG/RANKL/RANK pathway activities by CMD, the current data preliminarily explain the protective function of CMD against various disorders.

In conclusion, our study reported for the first time the treatment effects of CMD on DOP and that the effects were associated with activation of the netrin-1/DCC/UNC5B pathway. However, the current study failed to elaborate the potential dose-dependent effect of CMD, and its interaction with netrin-1/DCC/UNC5B pathways was only implicitly demonstrated. Additionally, the current study employed a type I diabetes model, while most diabetes patients were type 2 in clinic. Therefore, future studies with type 2 diabetes model should be performed with a more detailed experimental design by focusing on the dose-dependent effect of CMD as well as the direct interaction between CMD and netrin-1 or other targets.

## Data Availability

The raw data supporting the conclusion of this article will be made available by the authors, without undue reservation.

## Abbreviations

CMD, Cinnamaldehyde; DOP, diabetic osteoporosis; DM, diabetes mellitus; STZ, streptozocin; UNC5, uncoordinated 5; DCC, deleted in colorectal cancer; TRAP, tartrate-resistant acid phosphatase; OPG, osteoprotegerin; RANK, receptor activator of nuclear factor kappa B; RANKL, RANK ligand; PTH, parathyroid hormone; BS/BV, bone surface/bone volume ratio; BV/TV, bone volume/tissue volume ratio; Tb, Th trabecular thickness; Tb, N trabecular number; Tb, Sp trabecular separation; H&E, Hematoxylin–eosin; RT-qPCR, Reverse transcription quantitative polymerase chain reaction; IHC, Immunohistochemistry.
